# Psychopharmacological treatment in dissociative identity disorder (DID)

**DOI:** 10.1192/j.eurpsy.2021.412

**Published:** 2021-08-13

**Authors:** R. Pinilla, C. Rodríguez Sabaté, B. Ordóñez Méndez, A. Sotillos, A. Hernández Mata

**Affiliations:** 1 Psiquiatría, Hospital de Getafe, Madrid, Spain; 2 Psychology, Hospital Universitario de Getafe, Getafe, Spain; 3 Psychiatry, Hospital Universitario de Getafe, Getafe, Spain; 4 Psiquiatría, Hospital Universitario de Getafe, Getafe, Spain

## Abstract

**Introduction:**

Patients with dissociative identity disorder (DID) present two or more identities. Although it is a widely questioned diagnosis, it is currently found in the main DSM-5 and ICD-10 diagnostic manuals. So far there is no standard psychopharmacological treatment for people with this pathology.

**Objectives:**

Describe the pharmacological treatment associated with the clinical evolution of a patient with DID.

**Methods:**

Follow-up was carried out in a mental health center for a year, undergoing psychopharmacological and psychotherapeutic treatment. The information is taken from the medical history.

**Results:**

The patient presents with anxious and depressive symptoms. She was referred from primary care with 50mg sertraline without response. Dose was increased to 100mg without response. New management started with desvenlafaxine 100mg, associated with lorazepam, partially reducing the symptoms. Later, the patient presented self-referentiality, sounding of thought, began to describe frequent memory losses and a rebound in anxiety-depression symptoms, increasing the dose of desvenlafaxine to 200mg and introducing haloperidol to 1.5mg. Three months later, she presented showing another identity, active, aggressive, pythiatic, without evident anxious symptoms that she previously presented in a marked way. Desvenlafaxine was adjusted to 100mg and haloperidol to 0.5mg every 12 hours. The patient evolved favorably, decreasing anxiety, depressive symptoms and memory loss, in addition to disappearing psychotic symptoms. This treatment was sustained, keeping the patient psychopathological and functional stability and allowing a psychotherapeutic approach.

**Conclusions:**

Treatment with desvenlafaxine and haloperidol was favorable to maintain clinical stability and allow other therapeutic approaches.High dose of antidepressant could favor the expression of another identity of the patient.

**Disclosure:**

No significant relationships.

**Keyword:**

antidepresive antipsicotic disociative memory-loss

